# New insights from the biogas microbiome by comprehensive genome-resolved metagenomics of nearly 1600 species originating from multiple anaerobic digesters

**DOI:** 10.1186/s13068-020-01679-y

**Published:** 2020-02-24

**Authors:** Stefano Campanaro, Laura Treu, Luis M. Rodriguez-R, Adam Kovalovszki, Ryan M. Ziels, Irena Maus, Xinyu Zhu, Panagiotis G. Kougias, Arianna Basile, Gang Luo, Andreas Schlüter, Konstantinos T. Konstantinidis, Irini Angelidaki

**Affiliations:** 1 Department of Biology, University of Padova, Via U. Bassi 58/b, 35121 Padua, Italy; 2 Department of Environmental Engineering, Technical University of Denmark, 2800 Kgs. Lyngby, Denmark; 3 CRIBI Biotechnology Center, University of Padova, 35131 Padua, Italy; 4 School of Civil & Environmental Engineering and School of Biological Sciences (Adjunct), Georgia Institute of Technology, 311 Ferst Drive, Atlanta, GA 30332-0512 USA; 5 Department of Civil Engineering, University of British Columbia, Vancouver, BC Canada; 6 Genome Research of Industrial Microorganisms, Center for Biotechnology (CeBiTec), Bielefeld University, Universitätsstr. 27, 33615 Bielefeld, Germany; 7 Hellenic Agricultural Organization DEMETER, Soil and Water Resources Institute, Thermi-Thessaloniki, Greece; 8 Shanghai Key Laboratory of Atmospheric Particle Pollution and Prevention (LAP3), Department of Environmental Science and Engineering, Fudan University, Shanghai, 200433 China

**Keywords:** Anaerobic digestion, Metagenome-assembled genomes, Biogas, Microbial community structure, Functional reconstruction

## Abstract

**Background:**

Microorganisms in biogas reactors are essential for degradation of organic matter and methane production. However, a comprehensive genome-centric comparison, including relevant metadata for each sample, is still needed to identify the globally distributed biogas community members and serve as a reliable repository.

**Results:**

Here, 134 publicly available metagenomes derived from different biogas reactors were used to recover 1635 metagenome-assembled genomes (MAGs) representing different biogas bacterial and archaeal species. All genomes were estimated to be > 50% complete and nearly half ≥ 90% complete with ≤ 5% contamination. In most samples, specialized microbial communities were established, while only a few taxa were widespread among the different reactor systems. Metabolic reconstruction of the MAGs enabled the prediction of functional traits related to biomass degradation and methane production from waste biomass. An extensive evaluation of the replication index provided an estimation of the growth dynamics for microbes involved in different steps of the food chain.

**Conclusions:**

The outcome of this study highlights a high flexibility of the biogas microbiome, allowing it to modify its composition and to adapt to the environmental conditions, including temperatures and a wide range of substrates. Our findings enhance our mechanistic understanding of the AD microbiome and substantially extend the existing repository of genomes. The established database represents a relevant resource for future studies related to this engineered ecosystem.

## Background

Anaerobic environments are ubiquitous in the biosphere. Some examples are the digestive tract of animals, paddy fields, wetlands and aquatic sediments. These environments play crucial roles in the degradation of organic matter and in the global carbon cycle. The anaerobic digestion (AD) process has great societal importance since it reduces our dependence on fossil fuels via its ability to generate methane within engineered bioreactors [1]. For these reasons, the AD process has been widely established as an efficient metabolic route allowing the conversion of organic wastes, agricultural residues and renewable primary products into energy and other valuable products, and accordingly has been promoted as a sustainable solution for resource recovery and renewable energy production underpinning the circular economy concept.

Methane is one of the most relevant end-products generated during the methanogenesis step of the AD process, and is produced by methanogenic *Archaea* [2, 3]. Methane production has been directly linked to the composition of the AD microbiome [4–6], and it is also under the control of microbial metabolism, which is in turn thermodynamically dependent on environmental parameters of the reactor [7]. The intimate connection between these parameters offers unique opportunities to improve process efficiency, which can be achieved through microbial selection or manipulation.

To improve the understanding of highly diverse and interconnected networks of AD microbiomes, several studies focused on the taxonomic and functional characterization of microbial communities originating from laboratory-scale biogas reactors [8–17] as well as from full-scale biogas plants [18–23] trying to connect microbiome compositions to prevailing process parameters [4, 24]. Other studies focused on the identification of the functional roles of particular species isolated from AD systems [25–27]. Cultivation-based approaches to isolate microorganisms from AD environments have yielded hundreds of novel species; however, this approach is limited since only the cultivable fraction of the microbiome is accessible. For deeply studied anaerobic environments such as the human gut microbiome, there are highly different reports regarding the cultivable fraction ranging from 20 to 95% of the operational taxonomic units [28]. To get insights into the genetic repertoire of non-cultivable biogas community members, metagenome sequencing, including assembly and binning strategies became highly valuable. Genome-centric metagenomic approaches have been developed to obtain large numbers of metagenome-assembled genomes (MAGs) across many different environments. However, a global meta-analysis study aimed at complementation and consolidation of AD microbiome MAG repositories is still lacking. Accordingly, it is necessary to integrate available metagenome sequence information for AD microbiomes in a joint endeavor addressing the compilation of genomes for common community members. It is predictable that this approach will yield genome information for various novel organisms residing in AD microbiomes and facilitate insights into their potential functions and life-style. New archaeal microorganisms, such as members of the *Verstraetearchaeota* [29] and *Bathyarchaeota* [30] were discovered on the basis of metagenome-assembled genomes, the latter ones being proposed to contribute to hydrolysis and subsequent fermentation of organic substrates within biotechnological biogas production processes [31]. MAG collections in combination with the corresponding metadata related to the AD process, will allow the implementation of a “Microbial Resource Management” platform [32] as basis for microbial community characterization. This resource will provide information on the genetic potential and the performance of microorganisms within AD environments. However, results from multidisciplinary cutting-edge -omics methodologies and bioinformatics tools have to be considered to recover functional information.

To address compilation of a large-scale AD microbial genome database, we present a comprehensive metagenome-centric analysis performed by incorporating nearly 0.9 Tbp of sequence data, representing a wide range of different biogas reactor systems from seven different countries. The use of a homogeneous assembly and binning workflow, associated with a de-replication strategy, identified the genomes of nearly 1600 distinct bacterial and archaeal species. In total, 134 publicly available metagenomes were analyzed to (1) provide a global reference database of genomes for future studies; (2) understand the relative microbial composition in different reactor systems; (3) evaluate the metabolic properties of the species present; (4) determine the importance of some crucial functional processes among samples and (5) estimate the replication index in different taxa. This resource provides the opportunity to holistically study the genetic potential and performance of taxa represented by MAGs and to relate their activities to changing environmental conditions and process parameters.

## Results and discussion

### Public metagenomes selection and data processing

**Fig. 1:**
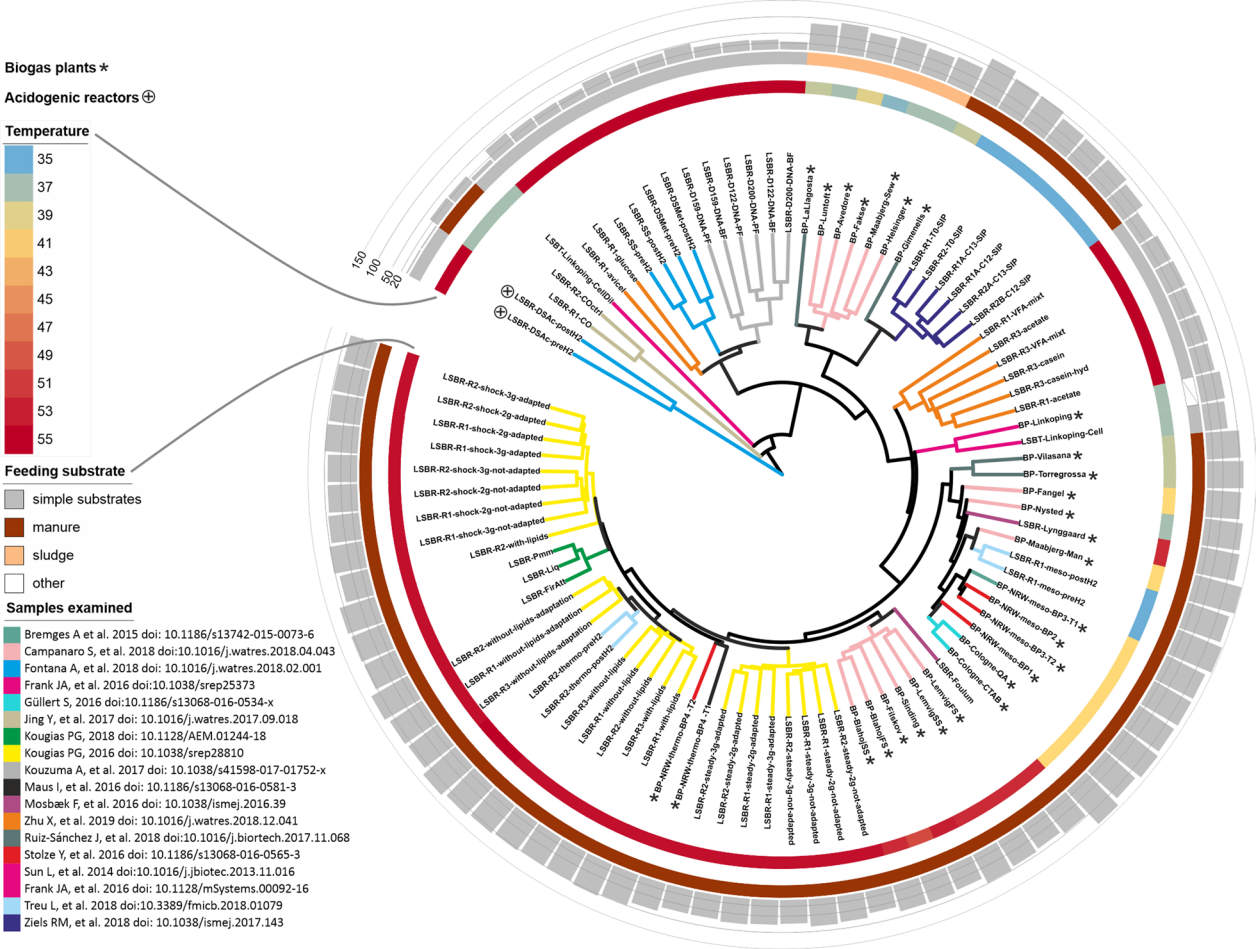
The tree is a representation of the β-diversity values determined from samples comparison. Reactor temperature and feeding substrates are reported in the external circles. Histogram graph in the external ring represents Fisher alpha diversity values

To get an overview of the AD microbiome, 18 experiments published between 2014 and 2019 were selected. These include 134 samples, some of them representing biological replicates (Fig. 1). Only experiments performed using Illumina sequencing technology have been considered in the present study, in order to facilitate the assembly and binning process. Among these datasets, both laboratory-scale- and full-scale biogas plants fed with a range of different substrates were considered, thus, the outcomes of the work reflect a broad spectrum of the microbiomes residing in such engineering systems. Most of the samples were collected from reactors operated in Denmark (68%), while others derived from Germany (9%), Canada (7%), Japan (7%), Spain (4%), Sweden (3%) and China (2%) (Additional file 1). Most samples were collected from laboratory-scale biogas reactors and batch tests, while other samples were obtained from 23 full-scale biogas plants located in Europe.

**Fig. 2:**
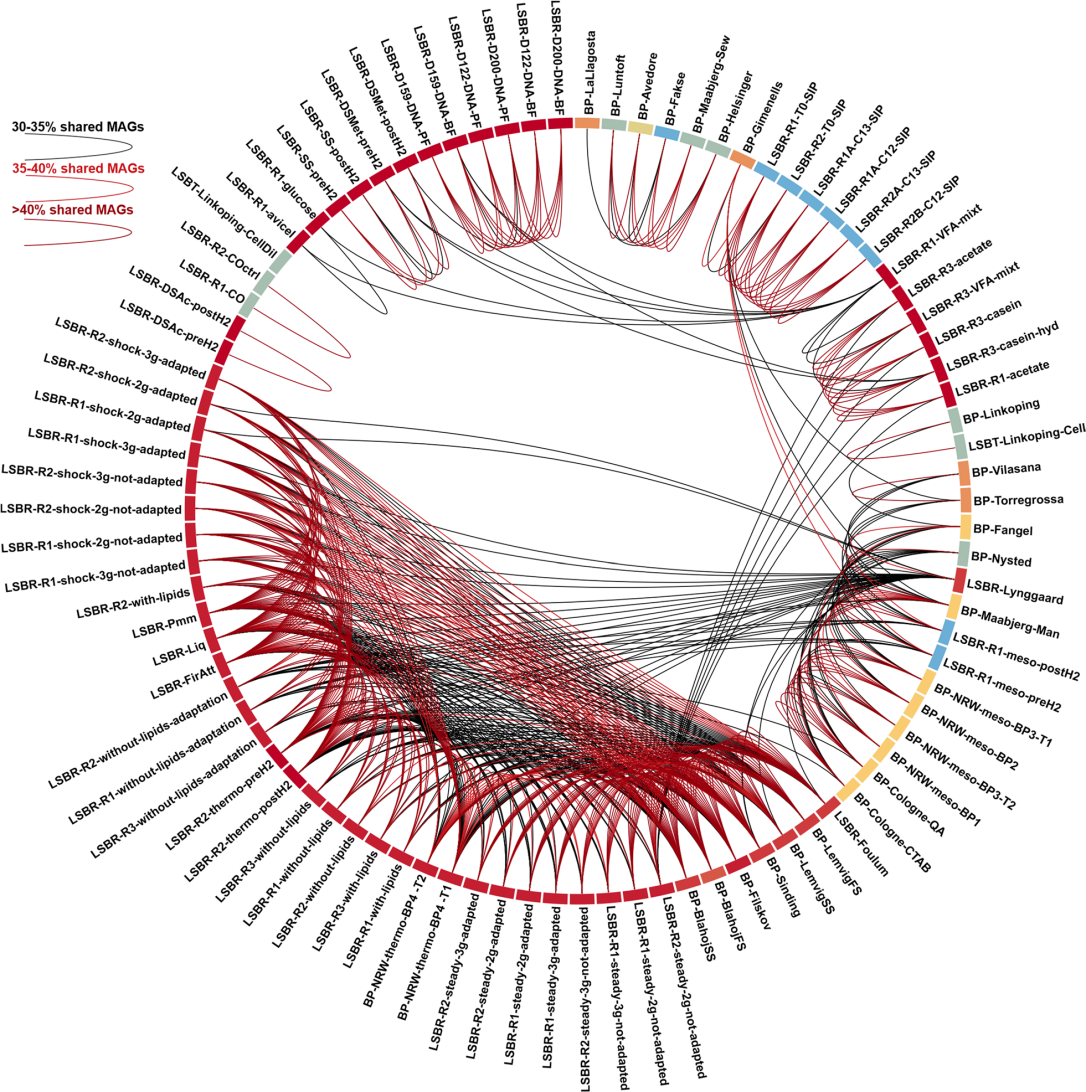
Representation of the MAGs fraction “shared” among samples. Arcs colored from black to dark red connect samples having increasing fractions of shared MAGs. Samples in the external circle are colored according to the temperature of the reactor

Microbial composition was initially determined considering unassembled reads, and this highlighted marked differences between samples, which were classified into 35 groups (details reported in Additional file 2). This microbial diversity is also clearly evident in Fig. 2, where different samples are connected with arcs having different colors depending on the fraction of common species.

**Fig. 3:**
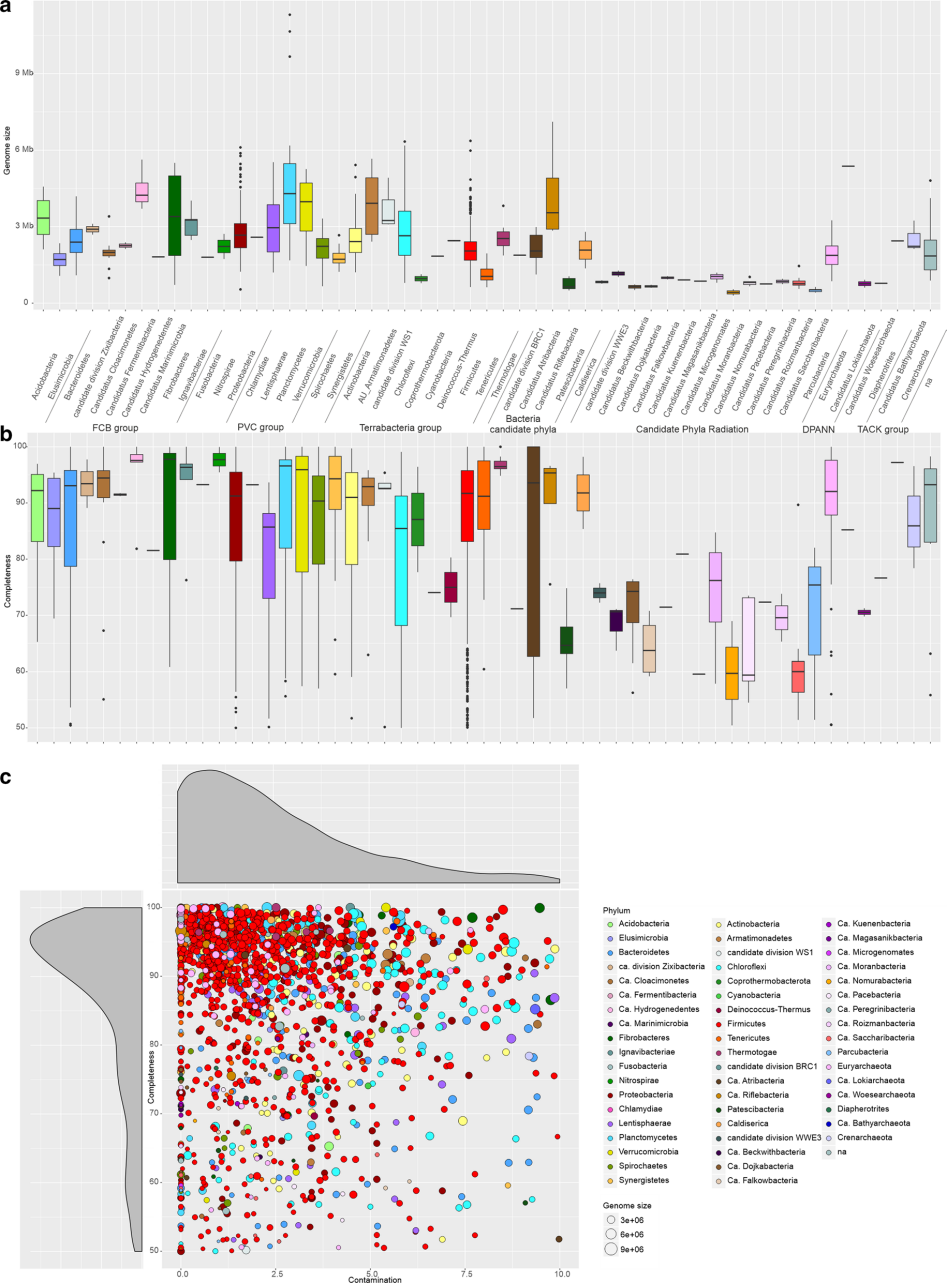
Box plots of genome size and completeness. **a** Genome size and **b** completeness of the 1635 selected MAGs. **c** Scatter plot reporting the completeness and contamination levels for each MAG (circle size is proportional to the genome length)

A subsequent binning approach was independently performed on each assembly of the 35 groups, leading to a total of 5194 MAGs (Table 1). Data regarding metagenomic assemblies and number of MAGs collected from the binning process are reported in detail in Additional file 3. Those MAGs featuring completeness (Cp) lower than 50% and/or contamination rates (Ct) higher than 10% were discarded. The remaining MAGs were de-replicated by means of the genome-aggregate ANI value reducing the number down to 1635 unique “species” (Table 1; Fig. 3; Additional file 4). By considering all 134 samples, on average 89% of the reads were consistently aligned on the 1635 MAGs, suggesting that the obtained dataset captured much of the available sequencing information. Results obtained were quite similar when only the HQ MAGs were selected. The degree of novelty of our study was determined performing a comparison with MAGs previously recovered from the AD environment [11, 33, 34] (https://biogasmicrobiome.com/). Our study showed an improvement in the quality (increased Cp and/or reduced Ct) of 75% of the MAGs already present in public repositories, and added 1228 “new species”, consistently improving the entire biogas microbiome (Additional file 5).

**Table 1:** Number of MAGs assigned to different categories according to their quality

	MAGs number before clustering (ANI)		MAGs number after clustering (ANI)
HQ MAGs Cp > 90%, Ct < 5%	1628	HQ MAGs selected from clusters	441
		HQ MAGs identified once	355
MHQ MAGs 90% > Cp ≥70%, 10% > Ct > 5%	1316	MHQ MAGs selected from clusters	170
		MHQ MAGs identified once	435
MQ MAGs 50% ≥ Cp > 70%, 10% > Ct > 5%	526	MQ MAGs selected from clusters	15
		MQ MAGs identified once	219
LQ MAGs Cp < 50%	1436		
Contaminated MAGs Ct > 10%	288		
Total	5194	Total	1635

*ANI* genome-wide average nucleotide identity

MAGs belonging to one cluster generated during ANI calculation are indicated as “Selected from clusters”, while MAGs not clustered at more than 95% ANI are indicated as “identified once”

### Structure of the microbial community

The analyses performed using MiGA estimated that a relevant fraction of the genomes belong to taxonomic groups for which genomes of type material are not present in the NCBI genome database. More specifically, 0.2% of MAGs cannot be assigned to known phyla, 11.6% to known classes, 69.7% to orders, 71.3% to families, 92.1% to genera and 95.2% to species. This evidenced that the present genome-centric investigation allowed to fill-in a notable gap in the knowledge of the AD microbial community. A dedicated project was established to allow the recovery of both genome sequences of MAGs and their taxonomic assignment “http://microbial-genomes.org/projects/biogasmicrobiome”.

**Fig. 4:**
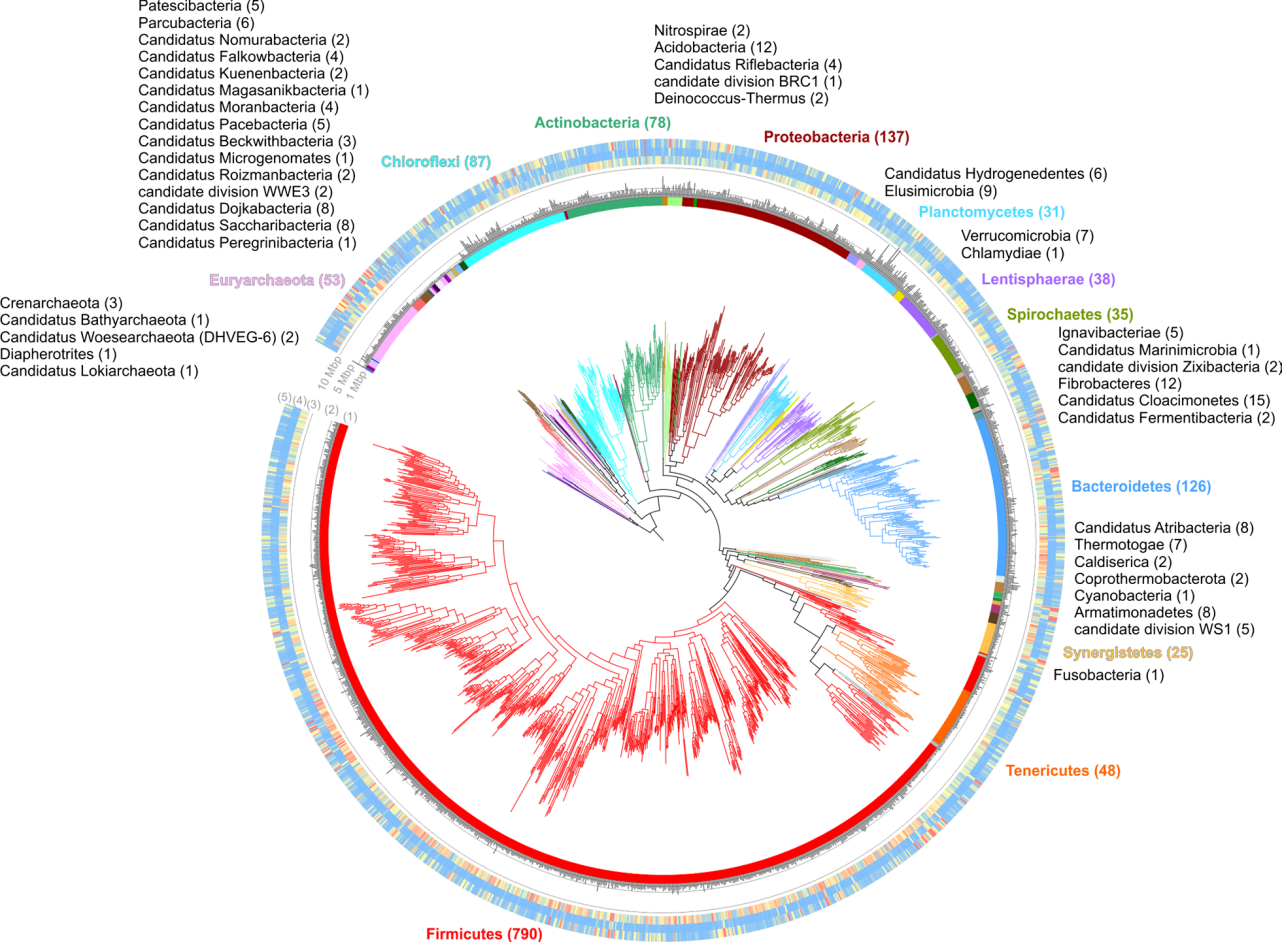
MAGs taxonomic assignment. The maximum likelihood tree was inferred from the concatenation of 400 taxonomic informative proteins and spans a de-replicated set of 61 archaeal and 1574 bacterial MAGs. External circles represent, respectively: (1) taxonomic assignment at phylum level, (2) genome size (bar plot), (3) heatmap representing the number of experiments where each MAG had abundance higher than 0.001% (from blue 0% to red 10%), (4) average abundance (from blue 0% to red 10%) and (5) maximum abundance determined among the entire set of experiments (from blue 0% to red 10%)

In addition, to determine the taxonomic position of the MAGs, a procedure based on four different evidences was used (Additional file 2). Only 69 out of 1635 MAGs were assigned to known species based on ANI comparison performed considering the genomes deposited in NCBI (https://www.ncbi.nlm.nih.gov/genome/microbes/) (Additional file 4). Furthermore, the vast majority of obtained MAGs (1574) were assigned to the domain *Bacteria* and only 61 to *Archaea*, and distributed over 55 different phyla as reported in Fig. 4. However, our data are similar to those previously obtained using marker gene based analysis [35], in fact, the vast majority of species were classified as belonging to the phylum *Firmicutes* (790 MAGs), followed by *Proteobacteria* (137 MAGs) and *Bacteroidetes* (126 MAGs). The bacterial phylum *Firmicutes*, which is the most abundant taxon within the biogas microbiome, varied between 1.3% and 99.9% of the microbial community (Additional file 2: Figure S1 and Additional file 6). In almost 40% of all samples analyzed, *Firmicutes* was not the dominant taxon, but *Bacteroidetes*, *Coprothermobacter*, *Actinobacteria*, *Thermotogae* and *Chloroflexi* become prevalent reaching up to 85% relative abundance within the microbiome. Interestingly, in reactors where none of the previously mentioned taxa were dominant, microbial species belonging to candidate phyla radiation (CPR) and to other candidate taxa reached high relative abundances, as was the case for *Candidatus* Cloacimonetes (15.7%), *Ca.* Fermentibacteria (16.4%), *Ca.* Roizmanbacteria (19%) and *Ca.* Saccharibacteria (16.4%) (Additional file 6). The high relative abundance of yet-uncultivated taxa suggests that they may play an important role in the microbial community. Some species associated to CPR were identified by our study and were tentatively assigned to *Saccharibacteria* (8 MAGs) and *Dojkabacteria* (8 MAGs), *Microgenomates* (1 MAG) and *Peregrinibacteria* (1 MAG).

Regarding the methanogenic community, it was shown that the AD microbiome is almost exclusively represented by phylum *Euryarchaeota* (53 MAGs).

### Influence of environmental conditions on the microbiome composition

It was shown that the applied environmental conditions (e.g., temperature), or the design of the reactors (e.g., biofilm), greatly determine the microbial diversity and properties of this ecosystem. For instance, the “*Bacteria/Archaea*” ratio, which has a median value of ~ 14, was highly variable (Additional file 2: Figure S2). Besides the acidogenic reactors, where the methanogenic process was undetectable (i.e., “LSBR-DSAc-preH2” and “LSBR-DSAc-postH2”), it was concluded that in 7.7% of all samples archaeal abundance was lower than 1% and consequently “*Bacteria/Archaea*” ratio exceeded 100. However, *Archaea* were predominant in several reactors analyzed in this study and in 3% of all samples, their abundance exceeded that of *Bacteria*, with a ratio of ~ 0.5 in a biofilm sample collected from a reactor fed with acetate (“LSBR-D200-DNA-BF”). Acetate is a very important “methanogenic substrate” and it can be directly converted to methane by acetotrophic Archaea. Thus, a dominance of *Archaea* in the microbial community is a reasonable finding, as evidenced in some samples of the present study. A complex combination of factors, such as the presence of biofilm, is probably contributing to this unbalanced proportion of the “*Bacteria*/*Archaea*” ratio. Considering only biogas plants, the ratio is kept within a narrower range, but still it is very flexible (from 470 in Nysted to 3.4 in Vilasana) (Additional file 2: Figure S2).

Furthermore, we calculated the variation in abundance for each MAG across the AD samples, along with their taxonomic assignment. The number of MAGs in each sample was estimated considering as “present” those with abundances higher than 0.001%. This analysis revealed that the microbial community composition was highly variable depending on the origin of each AD sample as a consequence of the reactor operation, performance, and influent feedstock (Figs. 1, 2 and Additional file 2: Figure S3). The number of detectable species in the microbiome ranged between 79 (Fisher alpha diversity 4.4) and 1213 (Fisher alpha diversity 133.8) (Additional file 7). According to previous findings [6, 9], thermophilic reactors have a lower number of species than mesophilic (*p* < 0.001). Among the thermophilic reactors in this study, those characterized by a very high number of species were fed with manure or a mixture of manure and agricultural feedstocks, while those having fewer species were fed with simplified substrates such as cheese whey, acetate or glucose (*p* < 0.001). This suggests that the AD process can be supported by less than 100 species when the feedstock is mainly consisting of a single compound. On the contrary, degradation of complex substrates (such as sewage sludge or manure) requires the cooperation of a large cohort of microbes including more than 1000 species. Analysis of the MAGs shared among different samples (Fig. 2) revealed that thermophilic reactors tend to share more species than mesophilic systems, which could be due to the selective pressure imposed by the high growth temperature. Despite feedstock is the primary determinant of community structure, it was previously demonstrated that the initial inoculum plays a major role, lasting for months even after feed changes [36]. Additionally, feedstock contributes to the community composition in terms of immigrant microbes, which are partially involved in shaping the final microbiome.

Cluster analysis was performed both at individual MAG abundance level and at sample level (Additional file 2: Figure S3) in order to verify MAGs and samples having similar abundance profiles, respectively. This allowed the assignment of MAGs to two main groups: “G1” includes mostly *Chloroflexi* and *Bacteroidetes*, while “G2” includes mostly *Firmicutes*. Sample clustering revealed three main groups, “C1” including reactors fed with sewage sludge, “C2” those fed with “simplified substrates” and “C3” fed with manure only. A similar classification is shown in Fig. 1, indicating that the temperature and feeding substrate were the main driving forces of the AD microbiome diversification [3, 35, 37, 38]. Furthermore, the principal coordinates analysis (PCoA) performed considering the microbiome composition originating from different AD environments revealed a clear separation of samples in three groups, one formed by thermophilic reactors fed with a mixture of carbohydrates and LCFA, one formed by thermophilic reactors fed with acetate and lactose and the third one represented by mesophilic samples (Additional file 2: Figure S4 A–C). This is in agreement with previous findings [3, 4] showing mostly specialized microbial communities depending on the temperature regime. The high heterogeneity in metadata accompanying the experiments evidenced the importance of establishing common guidelines regarding the parameters that have to be recorded during AD process. These standards will simplify the comparison among projects and will allow the correlation between metadata and microbial composition.

Considering a concept of “core microbiome”, meaning that some species are present in the anaerobic digestion microcosm independently of the applied environmental parameters, we identified only few MAGs in multiple samples (Additional file 2: Figure S3; Additional file 8). By considering the highly abundant MAGs (more than 1% relative abundance), only 25 were present in more than 10% of the samples, while 1246 were considered as low abundant (lower than 1%) (Additional file 2: Figure S5). Among the 25 abundant MAGs, four methanogenic *Archaea* were identified, namely the *Candidatus* Methanoculleus thermohydrogenotrophicum AS20ysBPTH_159, *Methanosarcina thermophila* AS02xzSISU_89, *Methanothrix soehngenii* AS27yjCOA_157 and *Methanoculleus thermophilus* AS20ysBPTH_14. The remaining 21 MAGs were assigned to the phyla *Firmicutes* (14 MAGs), *Bacteroidetes* (2 MAGs), *Synergistetes* (2 MAGs), *Thermotogae* (1 MAG) and *Coprothermobacterota* (1 MAG). Interestingly, *Defluviitoga tunisiensis* AS05jafATM_34, one out of seven MAGs of the phylum *Thermotogae* identified in this study, was present at high abundance (average 2.1%; maximum 58.9%). Widespread identification of this species in reactors suggests its central role in thermophilic AD system possibly associated to specific metabolic potential related to saccharide, polyol, lipid transport systems (Additional file 9) and hydrogen production [39]. Analysis of the low abundant MAGs (threshold 0.001%), revealed that 94% of these taxa were present in more than 10% of the samples, and the phyla statistically over-represented in this group were *Chloroflexi*, *Elusimicrobia*, *Firmicutes* and *Plantomycetes* (*p* < 0.01). This finding indicates that many MAGs are widespread in the global AD microbiome, but they are present at very low relative abundances. Differently from other ecological niches (e.g., human gut) a “core microbiome” present in all the reactors was not clearly identified. However, the existence of distinct core microbiomes characterizing groups of reactors with similar characteristics (e.g., feedstock or temperature) is more realistic, as also previously hypothesized [35].

### Functional analysis of the microbiome

**Fig. 5:**
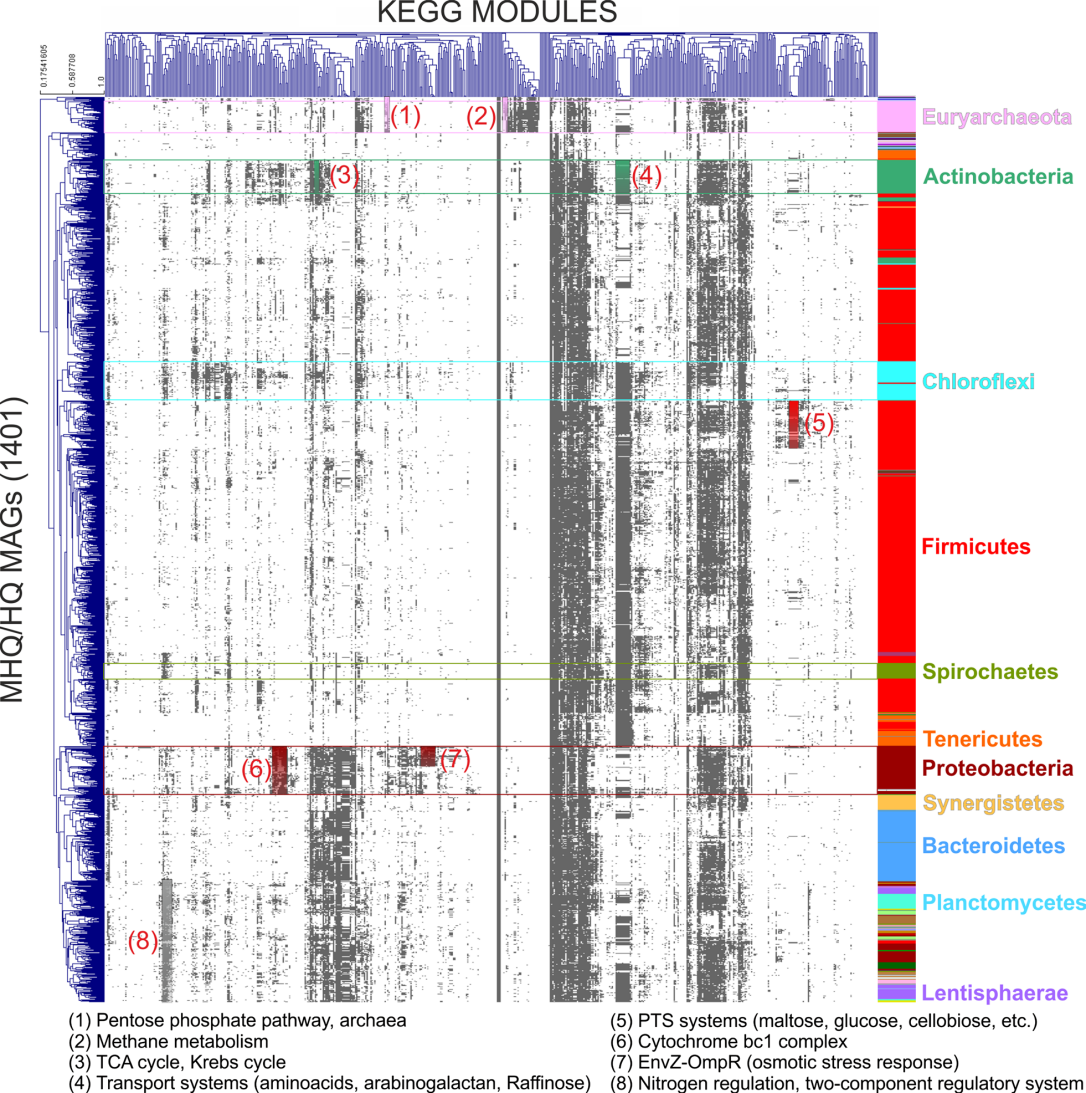
Hierarchical clustering of the “complete” and “1 bm” KEGG modules identified in the HQ and MHQ MAGs. In the right part of the figure taxonomic assignment is shown for the most represented phyla. KEGG modules specifically identified in selected phyla are highlighted

Metabolic pathway reconstruction and biological role interpretation of 1401 HQ and MHQ MAGs were performed by applying a collection of functional units, called KEGG modules. Analysis was performed on 610 modules, and identified that 76.2% of them are “complete” in at least one MAG, 10.1% have at best one block missing (1 bm) and 2.5% have at best two blocks missing (2 bm). In the following sections, only complete and “1 bm” modules will be considered. Modules distribution and completeness indicated that a very low number of them are widespread in MAGs, while the majority has a scattered distribution in terms of presence/absence (Fig. 5). Additionally, the association of many modules with some specific taxa is remarkable; in fact, a strong correlation between the clustering based on modules presence/absence and MAGs taxonomic assignment was found (Fig. 5; Additional file 10).

#### Main functions within the anaerobic digestion food-chain

Initial evaluation was focused on the identification of MAGs having a specific KEGG module. Considering both the complete and “1 bm” modules, only 15 “core modules” have been identified in more than 90% of the HQ-MHQ MAGs. These include for example “C1-unit interconversion”, “PRPP biosynthesis”, “glycolysis, core module involving three-carbon compounds”. Other 223 “soft core modules” were present in 10% to 90% of the HQ-MHQ MAGs. Finally, 289 “shell modules” have been identified in less than 10% of MAGs, including those associated with “methanogenesis”, “reductive citrate cycle” and “Wood–Ljungdahl (W–L)-pathway”. The high fraction of “soft core” and “shell” modules revealed a highly specialized microbial community, with a small number of species performing crucial functions such as methanogenesis. Results obtained revealed the presence of a small fraction of “multifunctional MAGs” (~ 1.6%) with more than 180 modules encoded. These microbes are mainly associated to specific taxa, and considering the HQ-MHQ MAGs, they represent 8.6% of the *Proteobacteria*, 14.3% of the *Chloroflexi*, 7.7% of the *Planctomycetes*. Thus, the AD microbiome typically comprises “oligofunctional” MAGs, which are characterized by the presence of less than 80 modules. Taxonomic distribution of the 89 HQ “oligofunctional” MAGs demonstrated that they were phyla-specific, representing 91.7% of the HQ *Tenericutes*, 32.2% of the HQ *Euryarchaeota* and 19.7% of the HQ *Bacteroidetes*.

#### Carbon fixation and methanogenesis

Particular attention was given to the modules associated with “methane metabolism”, and especially to the conversion of different substrates (carbon dioxide, acetate, methylamines and methanol) into methane. These modules were identified with different frequencies in the AD microbiome. Carbon dioxide reduction was identified in 29 MAGs, acetate conversion in 25 MAGs, methanol reduction in 40 MAGs and methylamine-methane conversion in 17 MAGs.

Apart from the fundamental role of methanogenesis in the AD system, the conversion of acetate, carbon dioxide and hydrogen can follow different pathways and can be strongly influenced by the environmental conditions. Practically, these flows are of particular interest for applying recent technologies, such as biomethanization or bioaugmentation. Considering the modules associated with carbon fixation, those encountered more frequently were the phosphate acetyltransferase–acetate kinase pathway (acetyl-CoA ⇒ acetate) identified in 1155 MAGs (82.4%) with 988 MAGs encoding the complete module, the reductive acetyl-CoA pathway (also called Wood–Ljungdahl pathway) identified in 86 MAGs (5.8%) with 52 encoding the complete module, and the reductive pentose phosphate cycle (ribulose-5P ⇒ glyceraldehyde-3P) identified in 128 MAGs (9.1%) with 42 encoding the complete module. The W-L pathway is present only in 0.49% of the microbial genomes deposited in the KEGG database; notably, this pathway is proven to be more common among the members of the AD microbiome. The taxonomic distribution of the 86 MAGs encoding the W-L pathway is mainly restricted to *Firmicutes* (75.6%), followed by *Chloroflexi* (9.3%), *Proteobacteria* (7%), *Euryarchaeota* (3.4%) and *Actinobacteria* (2.3%). Functional activity and syntrophic association with methanogens was previously reported for some of these species (e.g., *Tepidanaerobacter syntrophicus*, *Syntrophorhabdus aromaticivorans* and *Desulfitobacterium dehalogenans*) [40–42]. However, the vast majority was not previously characterized at the genome level, suggesting that potential syntrophic acetate oxidizer (SAO) or acetogenic metabolism are present in many unknown species. Most of the MAGs encoding the W-L pathway (putative SAO bacteria or acetogens) are rare in the microbiome and on average they do not exceed 1% of relative abundance. However, under certain conditions they can become dominant, as for example *Firmicutes* sp. AS4GglBPBL_6 (24.8% relative abundance in the Fangel biogas plant), *Firmicutes* sp. AS02xzSISU_21 (32% in reactor fed with Avicel) and *Firmicutes* sp. AS4KglBPMA_3 (12% in the Nysted biogas plant). This piece of information is quite useful for the design of bioaugmentation strategies targeting biogas reactors that are fed with nitrogen/ammonia rich substrates. Interestingly, the Fangel biogas plant showed a high total ammonia level during the sampling process (4.2 g/L) [43] (Additional file 1). This indicates that, despite SAO bacteria are usually present at low abundance, environmental parameters of the reactors can strongly influence their abundance and probably their activity. More specifically, high acetate concentrations can disturb acetoclastic methanogenesis leading to a shift towards SAO process coupled with hydrogenotrophic methanogenesis. Despite it is hard to classify the species mentioned above as SAO or acetogens, this result can provide a more accurate evaluation of the fraction of bacteria involved in acetate conversion and may support the delineation of a more accurate mathematical model for the AD process.

#### Relative abundance of KEGG modules

**Fig. 6:**
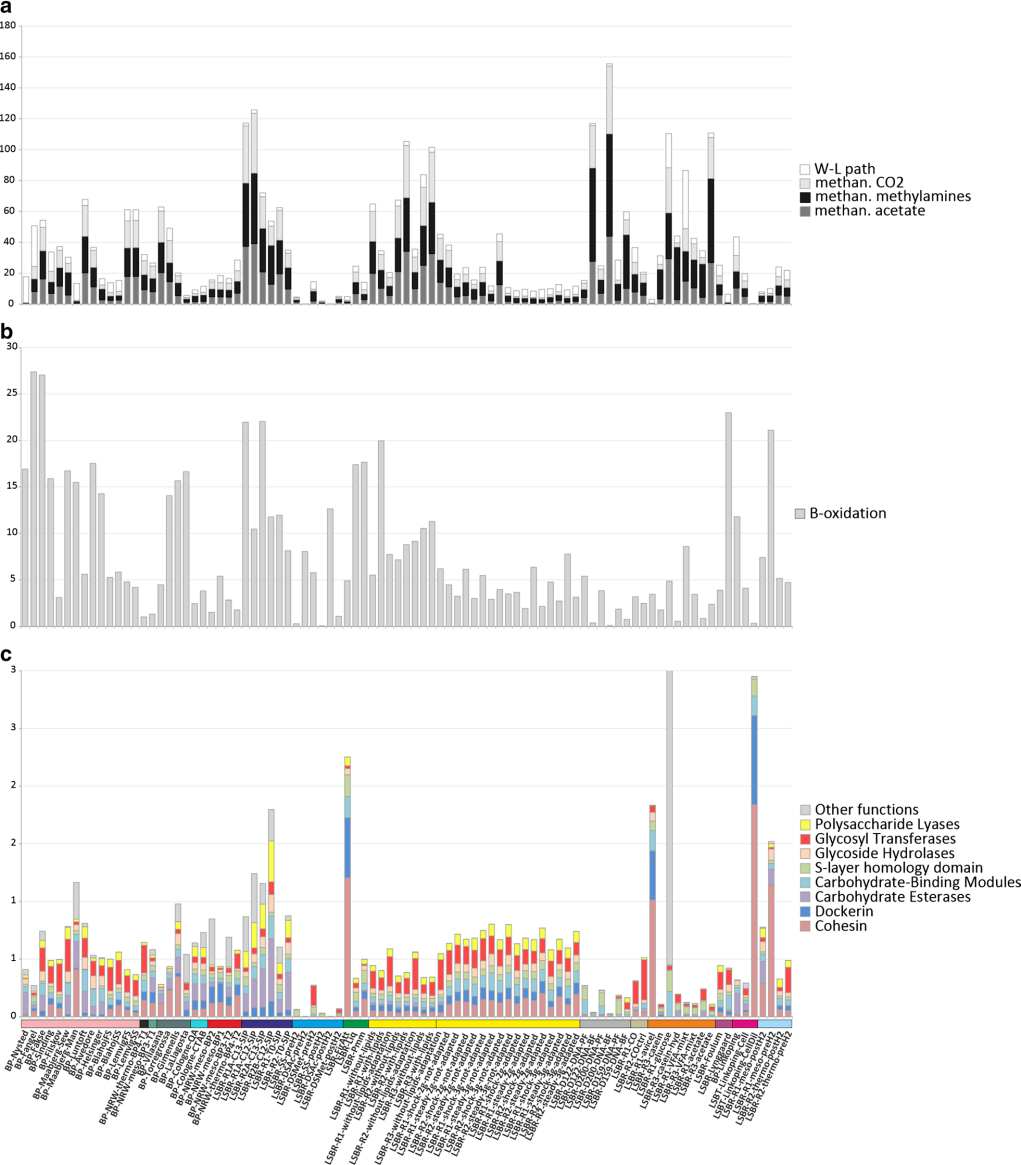
Representation of the relative abundance of relevant functional modules in the AD system: **a** “methanogenesis from CO_2_”, “acetate” and “methylamines” and “W-L pathway”, **b** oxidation pathway, **c** selected polysaccharide degradation modules. Bar graph was obtained for each sample by summing the relative abundance of all the HQ and MHQ MAGs encoding these “complete” and “1 bm” modules. Samples collected from biogas plants are in the left part of the figure (first 26 samples), while those derived from laboratory reactors or batch tests are shown in the right part

Considering the relative percentage of HQ MAGs in each condition, along with the completeness of KEGG modules, it was possible to estimate the relative abundance of each module in all samples (Additional file 11). Although measurements at the RNA/protein level are needed to have direct information on pathways activity, it is evident that different samples have highly variable representation of crucial KEGG modules (Fig. 6). It is noteworthy that the relative abundance of MAGs potentially associated to the hydrogenotrophic and acetoclastic methanogenesis is highly variable among samples. Particularly, in biogas plants characterized by low TAN (1.9–2 mg/L) (e.g., “BP-Gimenells” and “BP-LaLlagosta”), acetoclastic methanogenesis is favored and the ratio acetoclastic/hydrogenotrophic is 0.94 and 0.99, while in biogas plants where TAN is high (4–7 mg/L) (e.g., “BP-Vilasana”, “BP-Torregrossa” and “BP-Fangel”) the ratio acetoclastic/hydrogenotrophic is 0.16, 0.21, 0.02. Analyzing reactors where ammonia levels were reported, it was indeed found a significant correlation (R^2^ 0.62, p 9.3 E^−5^) between ammonia concentration and the “acetoclastic/hydrogenotrophic” ratio. Additionally, there is a high level of acetoclastic methanogenesis in reactors fed exclusively with acetate, such as “LSBR-D122-DNA-BF-Rep1”, “LSBR-D200-DNA-BF-Rep1” and “LSBR-R3-acetate”. Relative abundance of the methanogenic modules was found to be highly different among samples considered. As expected, it was close to zero in acidogenic reactors (pH < 5, “LSBR-DSAc-preH_2_” and “LSBR-DSAc-postH_2_”) and very high in reactors with acetate as feeding substrate (e.g., “LSBR-D200-DNA-BF” or “LSBR-R1-acetate”). The high abundance of methanogenic modules in the latter reactors can be correlated with the direct use of the substrate by acetoclastic methanogens, with a parallel reduction of the species encoding the W-L pathway.

#### Polysaccharides degrading functions

Cellulosic biomass in AD is represented by agricultural residues and dedicated energy crops, and is the most abundant carbon source [44]. In order to find the species involved in complex carbohydrate decomposition, MAGs featuring high enrichments in CAZymes (p < 1*e−5) have been selected for further analysis (Additional file 12). Globally, 490 HQ MAGs (35% of the total) are enriched in one or more CAZymes classes, evidencing that polysaccharide degradation is one of the most widespread functional activities in the AD system. Although polysaccharide degraders are frequently associated to *Firmicutes* (246 MAGs) and *Bacteroidetes* (68 MAGs), many other phyla were found to be enriched, and an involvement in polysaccharide degradation can be hypothesized for members of other taxa. For example, all MAGs belonging to the Candidatus *Hydrogenedentes*, the *Armatimonadetes*, 90% of the *Fibrobacteres*, 93% of the *Lentisphaerae* and 85% of the *Planctomycetes* are potentially involved in this process. Some members of the CPR taxa are also predicted as associated to carbohydrate degradation, such as Candidatus *Dojkabacteria*.

A tentative estimation of the relative impact of the polysaccharide degradation process in different samples (Fig. 6c) was obtained by considering the relative abundance of MAGs encoding genes for a specific function (e.g., “cohesin”, “dockerin”, or “Carbohydrate Esterases”). A few samples are dominated by polysaccharide hydrolyzing MAGs, (e.g., “LSBR-R1-avicel”), most probably because they were fed with substrates rich in cellulose, while generally the fraction is lower than 2%, particularly in biogas plants (Fig. 6c). This indicates that, despite the number of MAGs involved in polysaccharide degradation is high, the relative abundance of most species is low. This can be due to the presence of relative minor players in terms of abundance, but having high transcriptional activity; if they are highly active, they can enhance or trigger the metabolic processes of dominant members. However, this needs additional verification to be demonstrated.

### MAGs replication index

Analysis of MAGs provides insights into the genetic composition of non-cultivable biogas community members and enhances our understanding of their contribution to the AD process. Such analysis is able to provide knowledge related to the replication capacity of certain biogas-producing members. Although the results obtained have to be considered with caution, bacterial replication index offers information on the growth dynamics and life cycles of microbial species, which in turn can be an indicator of community composition and the in situ activity of different species within the sub-communities.

**Fig. 7:**
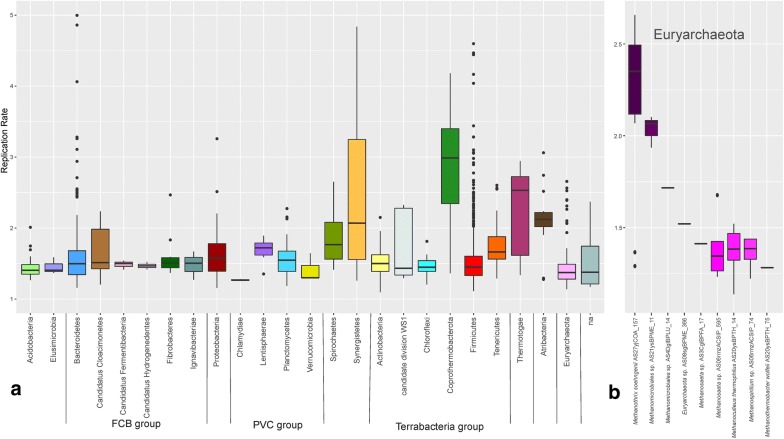
Box plots reporting the index of replication for some selected taxonomic groups. Index of replication. **a** Distribution of iRep values obtained for 538 MAGs belonging to each of the 25 phyla having at least three MAGs (“na” refers to taxonomically unassigned MAGs). **b** Distribution of iRep values obtained for Euryarchaeota. MAGs having only one value are reported as a horizontal bar

To determine the replication index of MAGs across multiple samples, the sequencing coverage resulting from bi-directional genome replication was used to calculate the index of replication (iRep) [45]. In total, 2741 measurements were obtained for 538 MAGs (Additional file 13). Considering the median iRep values determined in all different samples for each MAG, it was obvious that nearly 90% of species showed similar values between 1.1 and 2, and only 10% had values between 2 and ~ 4 and can be considered as “fast growing”. Among the fast growing species, there are microbial members of the poorly characterized phylum *Atribacteria* (*Atribacteria* sp. AS08sgBPME_53, iRep 2.9), and the candidate syntrophic species *Defluviitoga tunisiensis* AS05jafATM_34 (iRep 2.53) [39]. Results were obtained for 28 phyla evidencing that *Tenericutes*, *Spirochaetes*, *Atribacteria*, *Thermotogae*, *Synergistetes*, and *Coprothermobacterota* have on average high median iRep values (iRep 1.66, 1.77, 2.12, 2.53, 2.13, 2.99, respectively) (*p*-values 8.63E−10, 2.52E−04, 7.59E−04, 2.61E−05, 2.22E−11, 0.016), while *Euryarchaeota* and *Acidobacteria* have low values (1.37 and 1.41) (*p*-values 7.02E−05 and not statistically significant NSS, respectively) (Fig. 7a). *Euryarchaeota* species having multiple replication origins were 18 and have been excluded from the analysis (Additional file 2), however results should be treated with caution. MAGs belonging to the phyla *Bacteroidetes* and *Firmicutes* have similar (and low) median iRep values (both 1.52) except some outliers. Otherwise, iRep values assigned to *Synergistetes* and *Coprothermobacterota* are distributed over a wide range, but on average are higher than that of other phyla (2.12 and 2.99) (Fig. 7). The limited growth rate of some taxa, such as *Acidobacteria*, was also previously reported [46] and it was speculated that this property hampered their isolation. The high iRep values measured here for some known species also suggest that their isolation may be easier as previously assumed [47].

Finally, *Euryarchaeota* replication index was calculated (~ 1.52 on average) for 8 MAGs having different abilities in substrate utilization. Interestingly, while *M. soehngenii* was previously defined as a slow-growing methanogen specialized in acetate utilization [48], 7 out of 9 iRep results obtained for *M. soehngenii* AS21ysBPME_11 are higher than 2, while all the other *Archaea* had values between 1.2 and 2 (Fig. 7b). Findings reported for AS21ysBPME_11 indicate that, in a complex microbiome, growth rates can be very different compared to those determined for isolated species under laboratory conditions, possibly because of cooperative/syntrophic associations with other microbes, or difficulties in identifying the appropriate growth medium.

Our findings also suggest that duplication rates are dependent on metabolic properties of MAGs. Calculation of iRep values performed independently for MAGs encoding different KEGG modules evidenced that MAGs involved in polysaccharide degradation have quite low iRep values; this is more evident for microbes growing attached to plant material with cohesin/dockerin domains (iRep 1.41) (*p*-value 0.024). These species represent the so-called slow-growing cellulolytic microflora [49]. Species involved in “carbon fixation” (e.g., “reductive citrate cycle” or “W–L pathway”) have higher values (iRep 1.40; 1.53) (p-values 1.44E−08 and NSS, respectively). Additionally, iRep values were obtained for poorly characterized taxa such as *Atribacteria* and Candidatus *Fermentibacteria* (Fig. 7a), suggesting that most of the species are slow-growing members of the AD system, but with some exceptions such as *Atribacteria* sp. AS08sgBPME_53.

Availability of iRep values for a large number of species, and their association with functional roles of microbes can provide an estimate of the growth dynamics of species involved in particular steps of the AD food chain. Since nowadays mathematical models of the AD system are based on growth rates measured for a limited number of species, information obtained from iRep can provide a more generalized representation of microbial dynamics which can be included in simulations, reinforcing their predictive efficiency.

## Conclusions

The current comprehensive genome-centric assessment of the AD microbiome proves the great plasticity of this ecosystem upon variations on applied environmental conditions, such as reactor type, operational temperature and influent feedstock composition. The microbial adaptation is facilitated by the presence of multiple different microbial communities that have little to no overlap among them. Considering the abundant MAGs, only 25 were commonly identified in numerous samples. On the other hand, there are many other MAGs constituting a persistent, but low-abundant microbiome. Our findings related to metabolic pathways showed a partitioning of microorganisms according to their predicted substrate utilization capacities. Investigation of metabolic pathways suggested that some crucial processes, such as conversion of acetate to CO_2_, may be performed by a limited number of species. The high heterogeneity regarding protocols used for sample collection/processing and metadata registration evidenced that a common procedure is direly needed to obtain easily comparable datasets. By reconciling numerous metagenomics studies previously reported in the literature, this study suggests that the establishment of a global repository on microbial genome sequence information is of great importance for future studies and enhances our understanding of their contribution to the AD process.

## Methods

### Selection of samples and reads filtering

Illumina sequences were downloaded from Sequence Read Archive (SRA), MG-RAST or JGI Genome portal databases. Quality check and adaptors removal were performed using Trimmomatic (v0.33) and bbduk (version released Nov 2016) (https://jgi.doe.gov/data-and-tools/bbtools/). The composition of the feedstocks used in the different reactors was approximated using substrate information from various sources (Additional file 1). When available, metadata were taken from the publicly accessible description of the respective experiments or full-scale plant operation datasets. Otherwise, reactor feedstock compositions were estimated from the available literature, and were expressed in terms of carbohydrate, protein, lipid and VFA fractions relative to their total solid (TS) content.

### Assembly

Reads were assembled using Megahit (v1.1.1) with “−sensitive” mode for samples having less than 40 Gb of sequenced bases and with “–large” for the remaining [50]. Quality of the assemblies was determined using QUAST (v3.1) [51] and the results are reported in Additional file 8.

### Binning

Using MetaBAT 2 (v2.12.1) bam files were inspected and each assembly was binned using standard parameters [52]. Minimum size of scaffolds considered for MAGs generation was 1.5 kbp. MAGs were checked for completeness (Cp) and contamination (Ct) using the “Lineage_wf” workflow of CheckM (v1.0.3) [53] and the result obtained for each MAG was determined using the formula: CC3 = Cp − (Ct*3). Removal of contamination from MAGs was performed using RefineM (v0.0.23) [54]. Threshold values used for defining the quality level of MAGs and to assign them to the categories “High Quality” (HQ), “Medium–High Quality (MHQ), “Medium Quality” (MQ) and “Low Quality” (LQ) were defined according to the standards recently described, except for the introduction of the MHQ class (Table 1) [55].

### MAGs de-replication

MAGs obtained were de-replicated using Mash (v2.0) [56] on the entire genome sequences with very permissive parameters (0.05 Mash-distance, roughly equivalent to 0.95 ANI and 100/1000 Matching-hashes). Subsequently, a more precise analysis was performed applying the genome-wide Average Nucleotide Identity metric (ANI) using protein-encoding nucleotide sequences only [57]. MAGs were considered as belonging to the same species if they showed ANI value higher than 95% and reaching at least 50% of genome coverage for both strains (on at least one of the two comparisons, “MAG1 vs. MAG2” or “MAG2 vs. MAG1”). Details regarding the assembly and binning procedure are reported in Additional file 2.

### Taxonomic assignment

Taxonomic classification was determined for 1635 MAGs obtained after de-replication and belonging at least to the MQ level. This approach was carried out as described previously [4] and more details can be found in the Additional file 2. MAGs were classified by comparison against all taxonomically classified taxa of the NCBI Genome Database (prokaryotic section) using Microbial Genomes Atlas MiGA Online [58].

### MAGs coverage calculation and relative abundance

Filtered shotgun reads randomly selected from each sample were aligned back to the entire collection of MAGs. Ordered “bam” files were inspected using CheckM [53] to calculate both the fraction of reads aligned and the relative abundance of each MAG. Analysis was performed using all reads available for each sample and verified using a representative subsample of one million reads per sample. Results obtained using the two datasets of sequences were highly similar (Pearson correlation coefficient was > 0.999 on MAGs representing more than 0.001% of the population). Results obtained using one Mread per sample are reported in Additional file 8. The value (0.001%) was also defined as the arbitrary threshold for considering one MAG as “present in a specific sample”. Coverage values obtained for each MAG were clustered with MeV (v4.9.0) using Pearson correlation and average linkage [59]. The fraction of MAGs shared between different samples was visually represented using CIRCOS (v0.69) [60]. Alpha and beta diversity were determined from the file reporting the number of reads per MAG using Past (v3.21) [61]. The same tool was used for statistical tests and graphical plots.

### Gene finding and annotation

Gene annotation was performed using three different procedures: (1) rapid annotation using subsystem technology (RAST annotation server) [62]. These results were reported in a table for comparative purposes (Additional file 14). (2) KEGG annotation and modules completeness were determined using “KEGG Mapping/Reconstructmodule.py” (https://github.com/pseudonymcp/keggmapping). Software assigned to the KEGG modules the results obtained from diamond (v0.9.22.123) alignment; only results having max log e-value 1e−5, min bitscore 50, min identity 25 were recovered. Abundance of all the KEGG modules in each experiment was calculated with custom perl scripts (https://sourceforge.net/projects/perl-scripts-kegg/). Cluster analysis on “complete” or “1 bm” KEGG modules identified in HQ and MHQ MAGs was performed using MeV (v4.9.0) [59]. (3) Enzymes involved in carbohydrates utilization were annotated using the carbohydrate-active enzyme database (CAZy) annotation web server dbCAN (dbCAN-fam-HMMs.txt.v4) based on hmmscan. hmmscan-parser.sh was used to filter output file with default parameters (if alignment > 80aa, use E-value < 1e−5, otherwise use E-value < 1e−3; covered fraction of HMM > 0.3) (hmmer.org) [63] (Additional file 12). Abundance of specific functional classes was determined using hypergeometric analysis and p-values corrected using false discovery rate as described previously [64].

### MAGs replication rate

Considering the genome size and the total number of reads mapped on each MAG, the coverage of each MAG was determined using Bowtie 2 (v2.2.4). The MAGs having completeness higher than 90%, contamination lower than 5%, a number of scaffolds per Mbp lower than 175 and a coverage value higher than five, were selected in order to determine their index of replication (iRep) applying the iRep software [45]. Pairwise Wilcoxon rank sum test was performed (pairwise.wilcox.test in R software v3.4.4) and p-values were corrected with Bonferroni adjustment. The number of replication origins in archaeal genomes was inspected using Ori-Finder 2 software [65] and those having none or more than one were excluded from further analyses.

### Diversity indices, statistics and PCoA

β-diversity (pairwise sample dissimilarity, clustering method UPGMA) was calculated applying the ExpressBetaDiversity (EBD) software (v1.0.7) [66]. Statistical calculations (Mann–Whitney with Bonferroni correction for identification of taxa enriched in different groups and t-test for the comparison of the number of species in reactors fed with different substrate), diversity indexes (including for example Dominance, Simpson, Shannon H, Evenness, Fisher alpha, Berger–Parker, Chao-1) and β-diversity (pairwise sample dissimilarity, Whittaker) calculations were performed using past software (v3.21) [61]. PCoA was performed with past software using Bray–Curtis as distance measure; solely acidogenic reactors were excluded from the analysis due to their strongly different microbial composition.

## Supplementary information

**Additional file 1.** Metadata of the samples included in the present study. The composition of the feedstocks used in the different reactors was approximated using substrate information from various sources. When available, such data was taken from the publicly accessible description of the respective experiments or full-scale plant operation datasets. Otherwise, reactor feedstocks were estimated by a proportionality-based mixing technique: taking the characteristics of their individual constituents from available literature and combining them according to their fresh matter (FM) or volatile solid (VS) ratio in the feed.

**Additional file 2.** Supporting methods containing detailed process for assembly, binning and taxonomic assignment. This file includes also supplementary figures (S1 to S5) and Supplementary Table S1, with the number of replication origins identified in the archaeal genomes.

**Additional file 3.** Global statistics of the assemblies and binning.

**Additional file 4.** MAGs taxonomy. MAGs obtained after redundancy removal (based on ANI calculation) were assigned to the taxonomy using three different methods and results were subsequently combined.

**Additional file 5.** Comparison of MAGs with those reported in previous projects.

**Additional file 6.** Relative abundance of each taxa in the samples examined. Relative abundance of all the MAG having the same taxonomic assignment were combined in order to determine the relative abundance of each taxon as reported in columns D-CP. All the taxonomic levels from kingdom to genus were considered.

**Additional file 7.** Diversity indexes. Indexes were calculated for each sample in order to estimate the characteristics of the microbiome. Results were obtained starting from the files reporting the number of reads assigned to each MAG on each sample (subsampling all the samples to 1 million reads) and subsequently elaborated using PAST software. Colors reported in line 2 refer to the projects from which reads have been collected. Remaining colors were assigned in order to discriminate low and high values in a heatmap scale.

**Additional file 8.** MAGs coverage per sample. MAGs coverage was calculated on all the samples (average values were considered for samples collected in replicates).

**Additional file 9.** KEGG modules. Completeness of each KEGG module was reported for the high and medium-high quality MAGs.

**Additional file 10.** MAGs having KEGG modules. Number of MAGs having each KEGG module are reported. Only MAGs having complete and 1 block missing KEGG modules are considered. In column B is reported the total number of MAGs per each phylum (considering only high quality and medium-high quality MAGs). KEGG modules are reported in columns.

**Additional file 11.** KEGG pathways abundance in different microbial samples. Abundance of all HQ and MHQ MAGs having a complete or "one block missing" KEGG module were summed and the value is reported here as an estimate of the abundance of this pathway in each sample.

**Additional file 12.** Enrichment of CAZymes classes in MAGs. Number of proteins assigned to different classes and determined using dbCAN software. Only HQ and MHQ MAGs have been considered.

**Additional file 13.** Index of replication. iRep values were calculated for each MAG in all the samples considered. Average values were calculated for replicate samples. All samples are reported in column 2, while MAGs IDs and names are reported in columns A and B.

**Additional file 14.** Results obtained using RAST (Rapid Annotation using Subsystem Technology). MAGs genes were assigned to the function using RAST and the number of genes identified per each functional category (FL = first level; SL = second level) are reported. Functional categories are reported in column B, while MAGs are reported in rows (2 is MAG ID and 3 is MAG name assigned considering taxonomy).

## Data Availability

Shotgun sequences used were downloaded from SRA, EBI, DDBJ, GJI or MG-RAST and all the information associated to the projects are reported in (Additional file 1). All the MAGs sequences are available through the MiGA database under the project “http://microbial-genomes.org/projects/biogasmicrobiome” in https://biogasmicrobiome.env.dtu.dk/ and the MHQ and HQ were deposited in the NCBI database under the bioproject PRJNA602310.

## Abbreviations

MAG: Metagenome-assembled genomes
AD: Anaerobic digestion
Cp: Completenesses
Ct: Contamination
CPR: Candidate phyla radiation
PCoA: Principal coordinates analysis
WL: Wood–Ljungdahl
TAN: Total ammonia nitrogen
iRep: Index of replication
SRA: Sequence Read Archive
HQ: High quality
MHQ: Medium–high quality
MQ: Medium quality
LQ: Low quality
ANI: Average nucleotide identity
RAST: Rapid annotation using subsystem technology
KEGG: Kyoto Encyclopedia of Genes and Genomes
CAZy: Carbohydrate-active enZyme
