# Supplementary material for: New insights from the biogas microbiome by comprehensive genome-resolved metagenomics of nearly 1600 species originating from multiple anaerobic digesters
Source: Biotechnol Biofuels. 2020 Feb 24;13:25. doi: 10.1186/s13068-020-01679-y (PMC7038595; doi:10.1186/s13068-020-01679-y)
Supplement: Supplementary file 2 — Additional file 2. Supporting methods containing detailed process for assembly, binning and taxonomic assignment. This file includes also supplementary figures (S1 to S5) and Supplementary Table S1, with the number of replication origins identified in the archaeal genomes. [file 13068_2020_1679_MOESM2_ESM.pdf]

New insights from the biogas microbiome by comprehensive genome-resolved metagenomics of nearly 1600 species originating from multiple anaerobic digesters

Stefano Campanaro[1,3], Laura Treu[1,2,*], Luis M Rodriguez-R[4], Adam Kovalovszki[2], Ryan M Ziels[5], Irena Maus[6], Xinyu Zhu[2], Panagiotis G. Kougias[7], Arianna Basile[1], Gang Luo[8], Andreas Schlüter[7], Konstantinos T. Konstantinidis[4], Irini Angelidaki[2]

(1) Department of Biology, University of Padova, Via U. Bassi 58/b, 35121, Padova, Italy

(2) Department of Environmental Engineering, Technical University of Denmark, Kgs. Lyngby, DK-2800, Denmark

(3) CRIBI Biotechnology Center, University of Padova, Padova 35131, Italy

(4) School of Civil & Environmental Engineering and School of Biological Sciences (Adjunct) Georgia Institute of Technology 311 Ferst Drive, Atlanta, GA 30332-0512

(5) Department of Civil Engineering, University of British Columbia, Vancouver, British Columbia, Canada

(6) Bielefeld University, Center for Biotechnology (CeBiTec), Genome Research of Industrial Microorganisms, Universitätsstr. 27, 33615 Bielefeld, Germany

(7) Soil and Water Resources Institute, Hellenic Agricultural Organization DEMETER, Thermi-Thessaloniki, Greece

(8) Shanghai Key Laboratory of Atmospheric Particle Pollution and Prevention (LAP3), Department of Environmental Science and Engineering, Fudan University, 200433, Shanghai, China

* Correspondence and requests for materials should be addressed to L.T. (email: laura.treu@unipd.it) ; tel.: +39 0498276306

Supporting methods

Detailed process for assembly and binning

Reads obtained from biogas reactors inoculated with the same inoculum were co-assembled, as well as those collected from primary and secondary reactors of the same biogas plant. On contrary, samples derived from reactors using different inocula and those collected once from a specific biogas plant were assembled individually. Before performing the co-assembly of reads obtained from different reactors, the similarity of the microbial composition among different samples was verified running MetaPhlAn2 (v2.2.0) on one million unassembled reads, randomly collected from each sample [1]. This preliminary check confirmed that samples collected from the same reactor, or collected from reactors using the same inoculum had on average similar microbial composition. The high diversity between groups indicated the need for a separate assembly of each group in order to minimize computational requests, as well as to avoid co-assembly of different strains belonging to the same species, a process resulting in lower quality of the assembled MAGs [2].

Reads were assembled using Megahit (v1.1.1) with "--sensitive" mode for samples having less than 40 Gb of sequenced bases and with "--large" for the remaining assemblies [3]. After the assembly process, a trial alignment with Bowtie 2 program (v2.2.4) [4] was performed using 100,000 randomly selected reads per each sample in order to calculate the fraction of reads aligned on each assembly. This allowed the identification of all samples having a reasonable alignment rate on each assembly (higher than 25%) and to select them for the subsequent binning step. Samples having less than 25% aligned reads were considered as being not informative and not used to determine the coverage profile of the scaffolds. Based on these preliminary results, the number of experiments considered for coverage calculation and subsequent binning ranged from 11 to 89 depending on assembly.

After assembly and binning, contaminating scaffolds for each MAG were identified considering their genomic characteristics (GC content and tetranucleotide composition). After the filtering step performed with RefineM [5], the "CC3 value" [CC3=Cp-(Ct*3)] (where Cp is completeness and Ct contamination determined using checkM) of each MAG was calculated again leading to only 159 MAGs showing an improved "CC3 value" after contamination removal; all the remaining MAGs were maintained in their initial condition (without performing the filtering step).

During the redundancy removal, a single representative MAG was collected for each cluster. To determine the representative MAG, after ANI calculation, from each cluster of MAGs which belong to the same species, a representative one with the highest CC3 value was selected. These MAGs were classified in three groups according to their quality and contamination levels: High Quality "HQ" (Cp>90%, Ct<5%), Medium-High Quality "MHQ" (90%>Cp>=70%; 5%<Ct<10%) and Medium Quality "MQ" (70%>Cp>=50%; 5%<Ct<10%).

Details regarding taxonomic assignment

Taxonomic assignment reported in the text is reported in [6] with small modifications: (1) The highest priority for taxonomy assignment has been given to the ANI results obtained comparing MAGs with genomes from NCBI database. gANI calculation was performed as described in the main text comparing MAGs and the genomes downloaded from NCBI microbial genome database

(last accessed date: May, 2018). 56 MAGs showed an ANI value higher than 95% and more than 70% of genes in common with the reference species. Other 149 MAGs were also highly similar to known species deposited at the NCBI microbial genome database, but these reference genomes were not taxonomically assigned at species level. Other 38 MAGs had average similarity which was higher than 95%, but the percentage of common genes ranged between 50% and 70%. Furthermore, affiliation of these microbes to the genus level was doubtful. (2) Intermediate priority for taxonomy classification was given to MAGs encoding the 16S rRNA genes longer than 300 bp. The 16S rRNA genes were identified for each MAG with in-house developed perl script using Hidden Markov Models obtained from RNAmmer [7] and taxonomy assessment was determined using RDP classifier trained on SILVA 132 ribosomal RNA (rRNA) database [8]. Taxonomy results were compared with those obtained from ANI and from taxonomically informative proteins (PhyloPhlAn and CheckM, "step 3" below) [9,10]. Five discordant results were manually verified and corrected removing possibly misassigned 16S rRNA genes. (3) Results obtained from taxonomically informative proteins (PhyloPhlAn and CheckM) were used for taxonomic classification of the remaining MAGs. Finally, results obtained applying all three methods were compared with each other in order to discover discrepancies, which were identified and manually corrected only for the MAG *Candidatus* Fermentibacter daniensis_AS4DglBPLU_32. An additional verification was performed on MAGs assigned to CPR, DPANN and some other hypothetical taxa by selecting 5278 representative genomes from NCBI microbial genomes database as described previously [6], building a tree using PhyloPhlAn [9] and performing a manual inspection assisted by Dendroscope (v1.4) [11].

From the results obtained, 1,233 MAGs were taxonomically assigned using selected marker genes, an additional 212 MAG were characterized based on results obtained from 16S rRNA gene sequences, the taxonomy of the 121 remaining MAGs (mainly belonging to candidate taxa) has been refined by manual inspection of their placement into a phylogenetic tree as previously described. Only 69 out of 1,635 MAGs were assigned to known species based on ANI comparison performed considering the genomes deposited in NCBI (https://www.ncbi.nlm.nih.gov/genome/microbes/) (Data set S6).

Taxonomic assignment obtained from the combined evidences mentioned above (marker genes, 16S rRNA, ANI and manual inspection) was compared with that obtained from MiGA [12] and results obtained were in good agreement; the fraction of MAGs consistently assigned to already existing taxa varied from 68% (family) to 88% (genus) depending on the taxonomic level.

Two additional taxonomic analyses were performed using Bin Annotation Tool (BAT) [13] and GTDB-Tk toolkit [14]. Results are available in Additional File 4.

References

1. Truong DT, Franzosa EA, Tickle TL, Scholz M, Weingart G, Pasolli E, et al. MetaPhlAn2 for enhanced metagenomic taxonomic profiling. Nat. Methods. 2015;12:902–3.

2. Olm MR, Brown CT, Brooks B, Banfield JF. dRep: a tool for fast and accurate genomic comparisons that enables improved genome recovery from metagenomes through de-replication. ISME J. 2017;11:2864–8.

3. Li D, Liu C-M, Luo R, Sadakane K, Lam T-W. MEGAHIT: an ultra-fast single-node solution for

large and complex metagenomics assembly via succinct de Bruijn graph. Bioinformatics. 2015;31:1674–6.

4. Langmead B, Salzberg SL. Fast gapped-read alignment with Bowtie 2. Nat. Methods 2012;9:357–9.

5. Parks DH, Rinke C, Chuvochina M, Chaumeil P-A, Woodcroft BJ, Evans PN, et al. Recovery of nearly 8,000 metagenome-assembled genomes substantially expands the tree of life. Nat. Microbiol. 2017;2:1533–42.

6. Campanaro S, Treu L, Kougias PG, Luo G, Angelidaki I. Metagenomic binning reveals the functional roles of core abundant microorganisms in twelve full-scale biogas plants. Water Res. 2018;140:123–34.

7. Lagesen K, Hallin P, Rødland EA, Staerfeldt H-H, Rognes T, Ussery DW. RNAmmer: consistent and rapid annotation of ribosomal RNA genes. Nucleic Acids Res. 2007;35:3100–8.

8. Quast C, Pruesse E, Yilmaz P, Gerken J, Schweer T, Yarza P, et al. The SILVA ribosomal RNA gene database project: improved data processing and web-based tools. Nucleic Acids Res. 2013;41:D590-6.

9. Segata N, Börnigen D, Morgan XC, Huttenhower C. PhyloPhlAn is a new method for improved phylogenetic and taxonomic placement of microbes. Nat. Commun. 2013;4:2304.

10. Parks DH, Imelfort M, Skennerton CT, Hugenholtz P, Tyson GW. CheckM: assessing the quality of microbial genomes recovered from isolates, single cells, and metagenomes. Genome Res. 2015;25:1043–55.

11. Huson DH, Scornavacca C. Dendroscope 3: an interactive tool for rooted phylogenetic trees and networks. Syst. Biol. 2012;61:1061–7.

12. Rodriguez-R LM, Gunturu S, Harvey WT, Rosselló-Mora R, Tiedje JM, Cole JR, et al. The Microbial Genomes Atlas (MiGA) webserver: taxonomic and gene diversity analysis of *Archaea* and *Bacteria* at the whole genome level. Nucleic Acids Res. 2018;46:W282–8.

13. von Meijenfeldt FAB, Arkhipova K, Cambuy DD, Coutinho FH, Dutilh BE. Robust taxonomic classification of uncharted microbial sequences and bins with CAT and BAT. Genome Biol.. 2019;20:217.

14. Chaumeil P-A, Mussig AJ, Hugenholtz P, Parks DH. GTDB-Tk: a toolkit to classify genomes with the Genome Taxonomy Database. Hancock J, editor. Bioinformatics. 2019

Supplementary figures

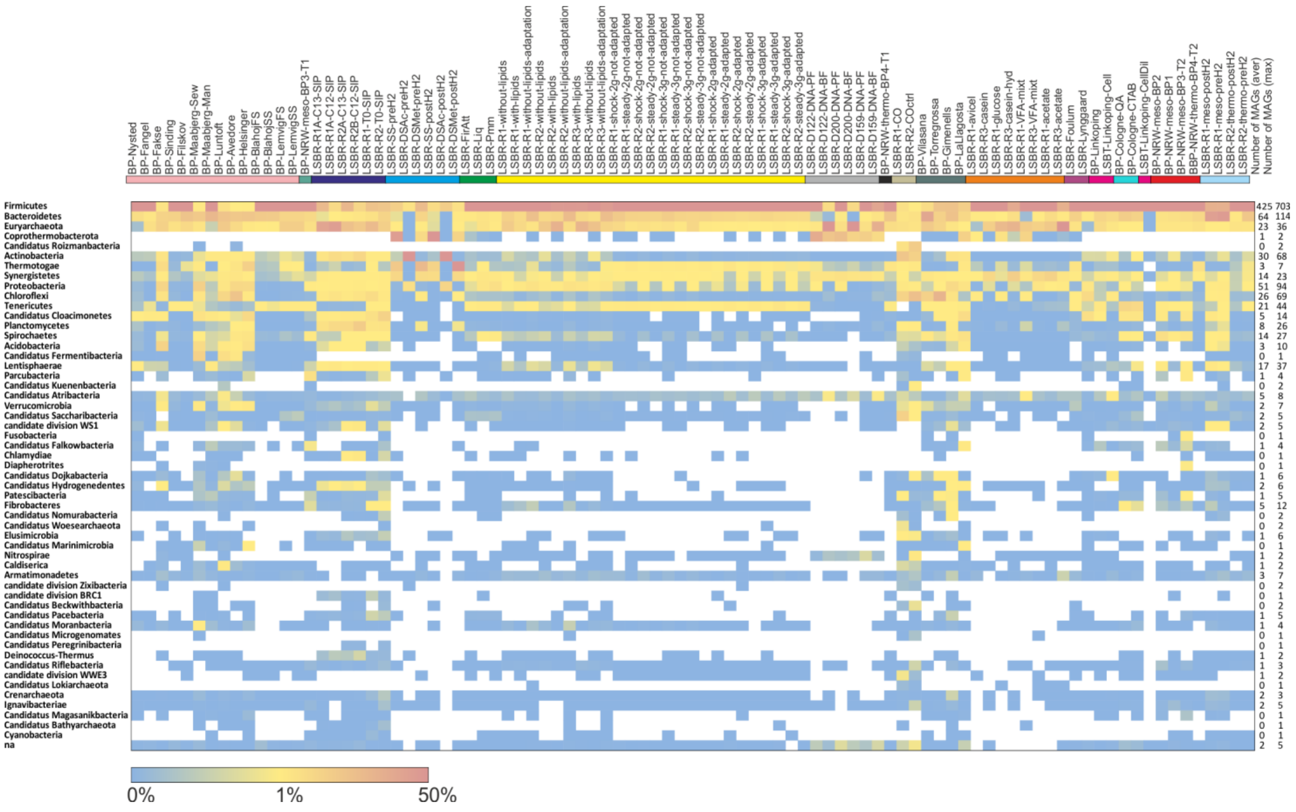

Fig. S1 Relative abundance of all the MAGs associated to the phyla identified is represented as a heatmap (see color scale at the bottom of the figure). On top of the figure color codes are associated to the different experiments considered. From top to bottom, phyla are ordered considering their average relative abundance in all the experiments.

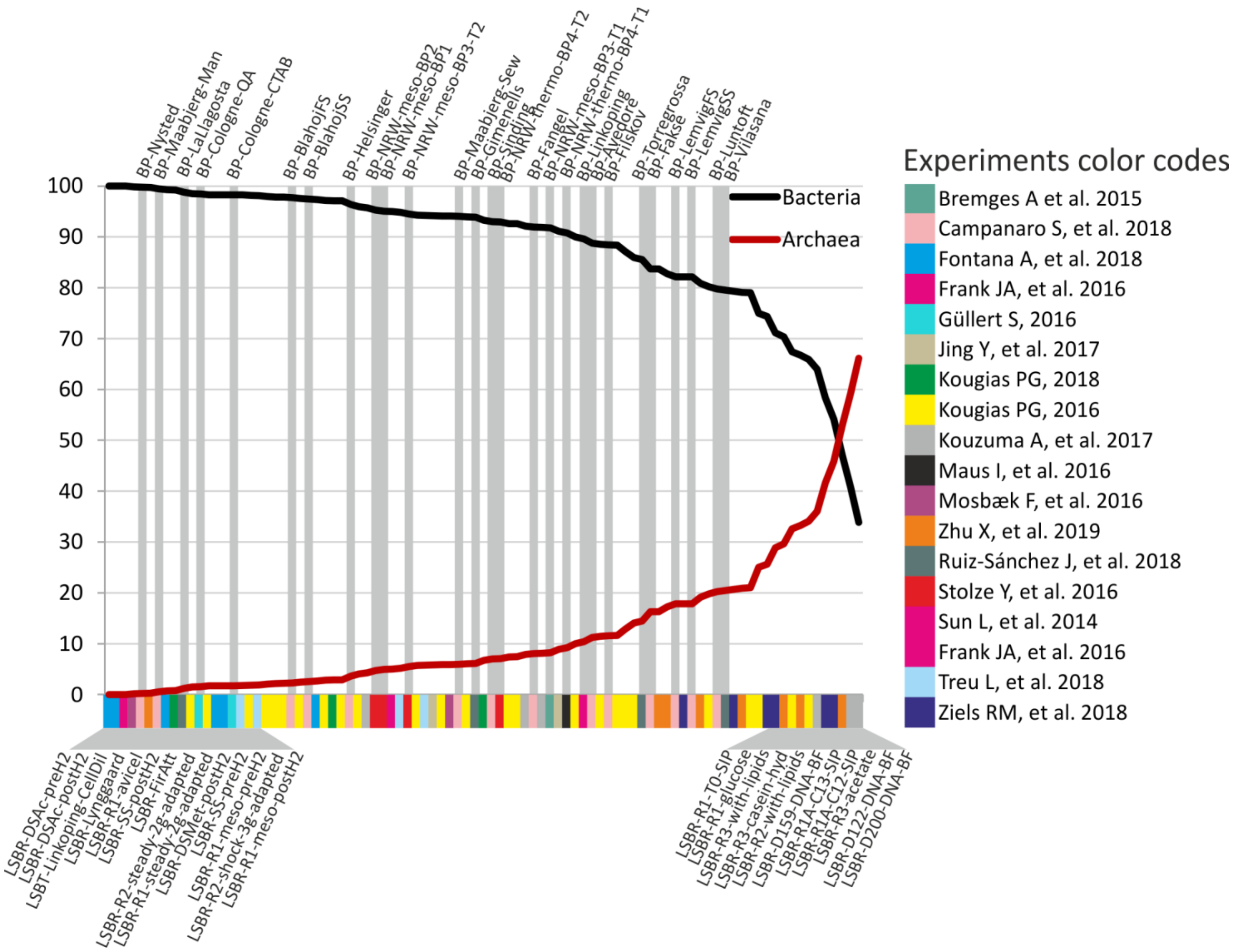

Fig. S2 Coverage values of all the MAGs calculated in each sample were used to estimate relative abundance of Archaea and Bacteria. Gray stripes in figure highlight samples collected from biogas plants.

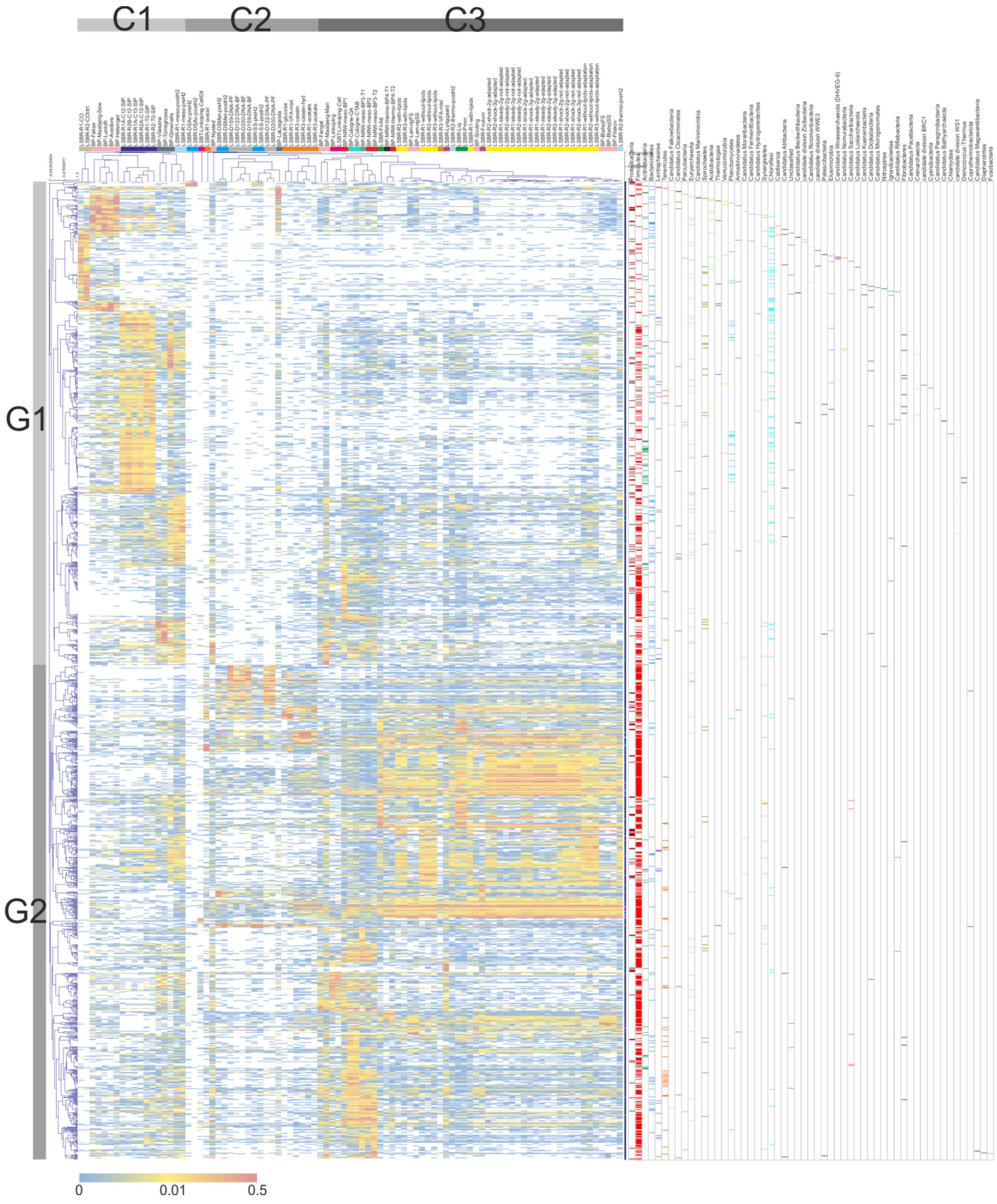

Fig. S3 Cluster analysis of the MAGs relative abundance values. White "cells" represent undetected MAGs, the remaining cells are reported according to a color scale with values increasing from blue to red (see color scale at the bottom). In the right part of the figure colors refer to the taxonomic assignment of the MAG at phylum level. Clusters of MAGs (G1, G2) and clusters of experiments (C1-C3) are discussed in the text.

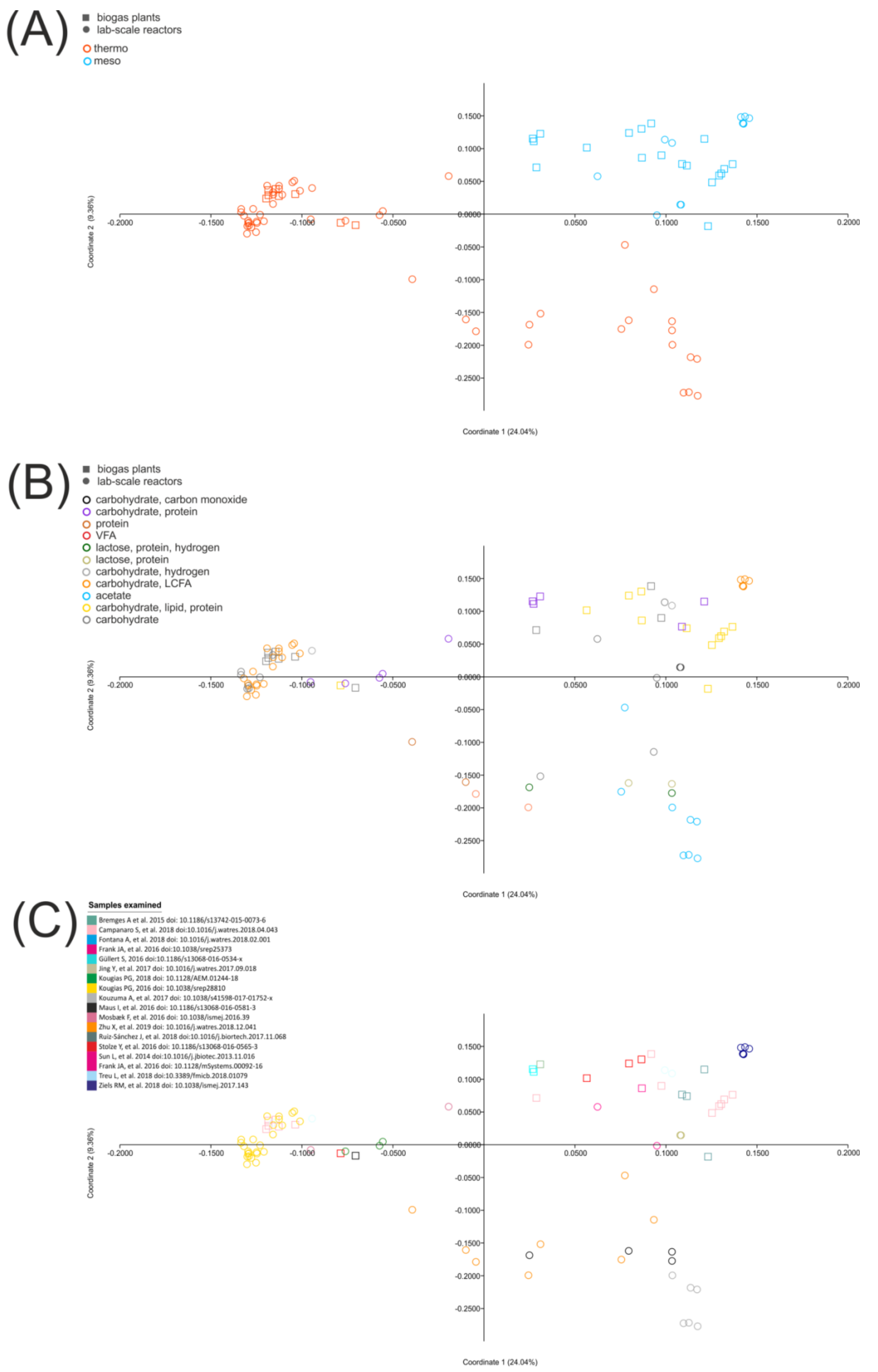

Fig. S4 Principal Coordinate Analysis (PCoA) performed considering MAGs abundance in the samples examined. Samples are colored according to the temperature (A), to the feedstock (B) and to the experiment (C). Feedstock composition was summarized according to the data reported in Supplementary data 1. Full-scale biogas reactors are reported as small squares, while laboratory-scale reactors as circles.

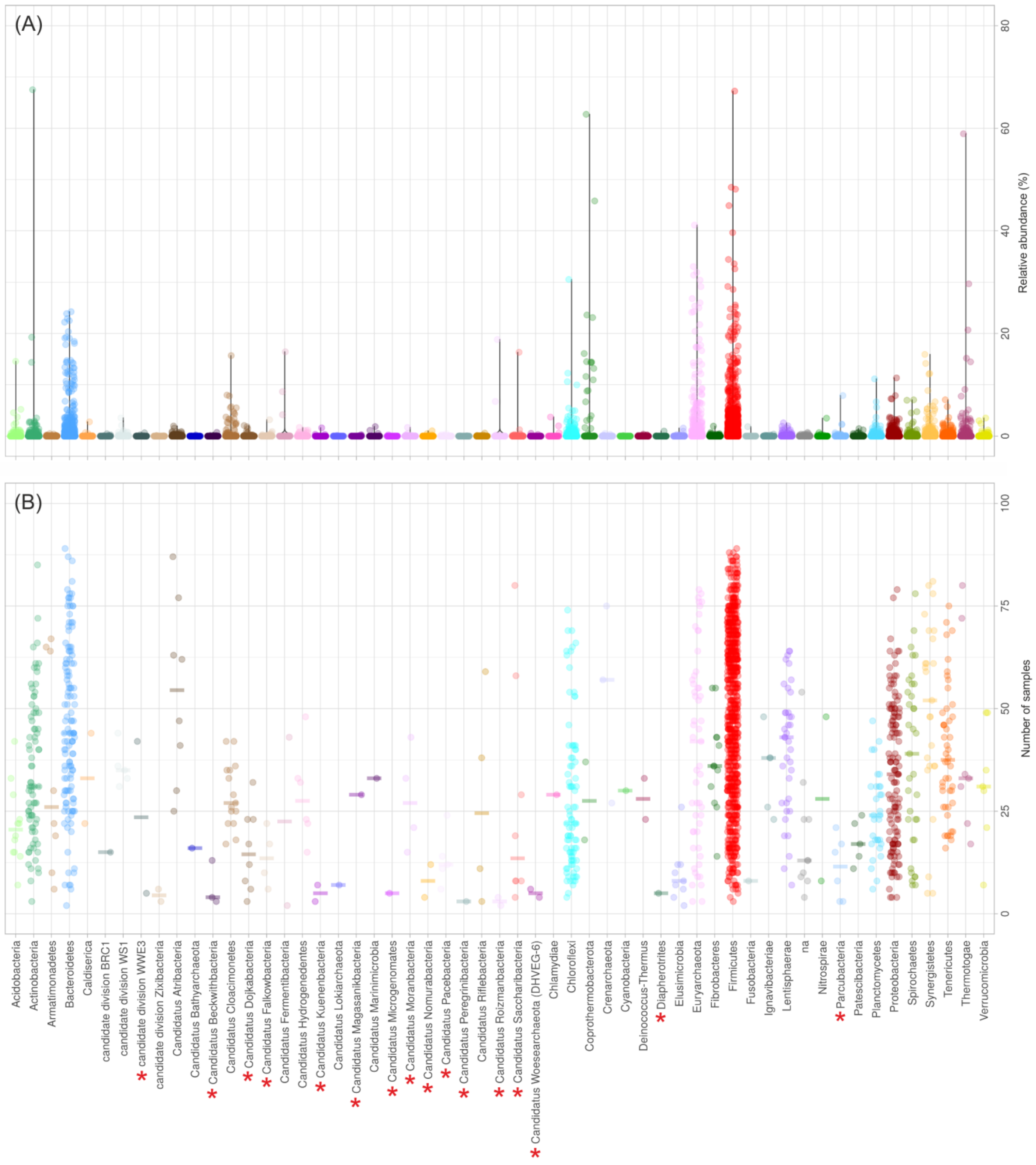

Fig. S5 MAGs relative abundance and their distribution in different samples. (A) Dots represent MAGs relative abundance in the samples examined. Dots are colored according to the taxonomic assignment of the MAGs at phylum level. (B) Number of samples where each MAG was identified at relative abundance higher than 0.001%. Each dot is representative of a single MAG and the number of samples where it was identified is reported in y axes (CPR are marked with asterisks).

Table S1. Number of replication origins identified in the archaeal genomes.

Species	Number of origins
Methanomicrobiales sp. AS06rmzACSIP_62	0
Candidatus Lokiarchaeota sp. AS27yjCOA_147	0
Crenarchaeota sp. AS06rmzACSIP_525	0
Diapherotrites Incertae Sedis sp. AS22ysBPME_79	0
Euryarchaeota sp. AS06rmzACSIP_150	0
Methanocorpusculum parvum AS07pgkLD_39	0
Methanomicrobiales sp. AS06rmzACSIP_125	0
Methanomicrobiales sp. AS06rmzACSIP_486	0
Methanomicrobiales sp. AS19jrsBPTG_12	0
Methanosarcina sp. AS05jafATM_20	0
Methanosarcina sp. AS10tlH2TH_61	0
Methanothermobacter sp. AS27yjCOA_93	0
Arc I group sp. AS22ysBPME_302	1
Arc I group sp. AS27yjCOA_88	1
Bathyarchaeota sp. AS06rmzACSIP_217	1
Crenarchaeota sp. AS06rmzACSIP_615	1
Euryarchaeota sp. AS06rmzACSIP_582	1
Euryarchaeota sp. AS08sgBPME_366	1
Euryarchaeota sp. AS21ysBPME_342	1
Euryarchaeota sp. AS22ysBPME_74	1
Euryarchaeota sp. AS23ysBPME_60	1
Methanoculleus thermophilus AS20ysBPTH_14	1
Methanomassiliicoccus sp. AS06rmzACSIP_208	1
Methanomicrobiales sp. AS06rmzACSIP_503	1
Methanomicrobiales sp. AS10tlH2TH_381	1
Methanomicrobiales sp. AS19jrsBPTG_18	1
Methanomicrobiales sp. AS21ysBPME_11	1
Methanomicrobiales sp. AS22ysBPME_16	1
Methanomicrobiales sp. AS22ysBPME_199	1
Methanomicrobiales sp. AS27yjCOA_146	1
Methanomicrobiales sp. AS2HglBPFA_19	1
Methanomicrobiales sp. AS4DglBPLU_14	1
Methanosaeta sp. AS06rmzACSIP_595	1
Methanosaeta sp. AS27yjCOA_18	1
Methanosaeta sp. AS27yjCOA_204	1
Methanosaeta sp. AS3CglBPFA_17	1
Methanothermobacter wolfeii AS20ysBPTH_75	1
Methanothermobacter sp. AS06rmzACSIP_462	1
Methanothrix soehngenii AS27yjCOA_157	1
Thermoplasmatales Incertae Sedis sp. AS10tlH2TH_373	1
Woesearchaeota DHVEG-6 sp. AS27yjCOA_155	1
Woesearchaeota DHVEG-6 sp. AS27yjCOA_212	1
Methanoculleus bourgensis AS23ysBPME_211	1
Arc I group sp. AS27yjCOA_5	2
Candidatus Methanoculleus thermohydrogenotrophicum AS20ysBPTH_159	2
Methanobacterium sp. AS08sgBPME_337	2
Methanomicrobiales sp. AS06rmzACSIP_125	2
Methanomicrobiales sp. AS06rmzACSIP_358	2
Methanomicrobiales sp. AS19jrsBPTG_32	2
Methanosarcina flavescens AS22ysBPME_46	2
Methanosarcina mazei AS10tlH2TH_287	2
Methanothermobacter sp. AS01afH2WH_50	2
Methanothermobacter sp. AS04akNAM_23	2
Thermoplasmatales Incertae Sedis sp. AS06rmzACSIP_286	2
Methanothermobacter sp. AS04akNAM_25	3

Euryarchaeota sp. AS22ysBPME_74	3
Methanosarcina thermophila AS02xzSISU_89	3
Methanosarcinaceae sp. AS23ysBPME_4	3
Thermoplasmatales Incertae Sedis sp. AS21ysBPME_157	3
Crenarchaeota sp. AS10tlH2TH_146	4
Methanothermobacter sp. AS05jafATM_76	4
